# Clinical utility of a rapid two-dimensional balanced steady-state free precession sequence with deep learning reconstruction

**DOI:** 10.1016/j.jocmr.2024.101069

**Published:** 2024-07-28

**Authors:** Katerina Eyre, Moezedin Javad Rafiee, Margherita Leo, Junjie Ma, Elizabeth Hillier, Negin Amini, Josephine Pressacco, Martin A. Janich, Xucheng Zhu, Matthias G. Friedrich, Michael Chetrit

**Affiliations:** a Research Institute, McGill University Health Centre, Montreal, Quebec, Canada; b GE HealthCare, Milwaukee, Wisconsin, USA; c Department of Diagnostic Radiology, McGill University, Montreal, Quebec, Canada; d Area19 Medical Inc., Montreal, Canada; e Division of Cardiology, McGill University, Montreal, Quebec, Canada

**Keywords:** Cardiac function, CMR, Deep learning reconstruction, Accelerated imaging, Diagnostic accuracy, Clinical utility

## Abstract

**Background:**

Cardiovascular magnetic resonance (CMR) cine imaging is still limited by long acquisition times. This study evaluated the clinical utility of an accelerated two-dimensional (2D) cine sequence with deep learning reconstruction (Sonic DL) to decrease acquisition time without compromising quantitative volumetry or image quality.

**Methods:**

A sub-study using 16 participants was performed using Sonic DL at two different acceleration factors (8× and 12×). Quantitative left-ventricular volumetry, function, and mass measurements were compared between the two acceleration factors against a standard cine method. Following this sub-study, 108 participants were prospectively recruited and imaged using a standard cine method and the Sonic DL method with the acceleration factor that more closely matched the reference method. Two experienced clinical readers rated images based on their diagnostic utility and performed all image contouring. Quantitative contrast difference and endocardial border sharpness were also assessed. Left- and right-ventricular volumetry, left-ventricular mass, and myocardial strain measurements were compared between cine methods using Bland-Altman plots, Pearson’s correlation, and paired t-tests. Comparative analysis of image quality was measured using Wilcoxon-signed-rank tests and visualized using bar graphs.

**Results:**

Sonic DL at an acceleration factor of 8 more closely matched the reference cine method. There were no significant differences found across left ventricular volumetry, function, or mass measurements. In contrast, an acceleration factor of 12 resulted in a 6% (5.51/90.16) reduction of measured ejection fraction when compared to the standard cine method and a 4% (4.32/88.98) reduction of measured ejection fraction when compared to Sonic DL at an acceleration factor of 8. Thus, Sonic DL at an acceleration factor of 8 was chosen for downstream analysis. In the larger cohort, this accelerated cine sequence was successfully performed in all participants and significantly reduced the acquisition time of cine images compared to the standard 2D method (reduction of 37% (5.98/16) p < 0.0001). Diagnostic image quality ratings and quantitative image quality evaluations were statistically not different between the two methods (p > 0.05). Left- and right-ventricular volumetry and circumferential and radial strain were also similar between methods (p > 0.05) but left-ventricular mass and longitudinal strain were over-estimated using the proposed accelerated cine method (mass over-estimated by 3.36 g/m^2^, p < 0.0001; longitudinal strain over-estimated by 1.97%, p = 0.001).

**Conclusion:**

This study found that an accelerated 2D cine method with DL reconstruction at an acceleration factor of 8 can reduce CMR cine acquisition time by 37% (5.98/16) without significantly affecting volumetry or image quality. Given the increase of scan time efficiency, this undersampled acquisition method using deep learning reconstruction should be considered for routine clinical CMR.

## Graphical abstract

**Figure:**
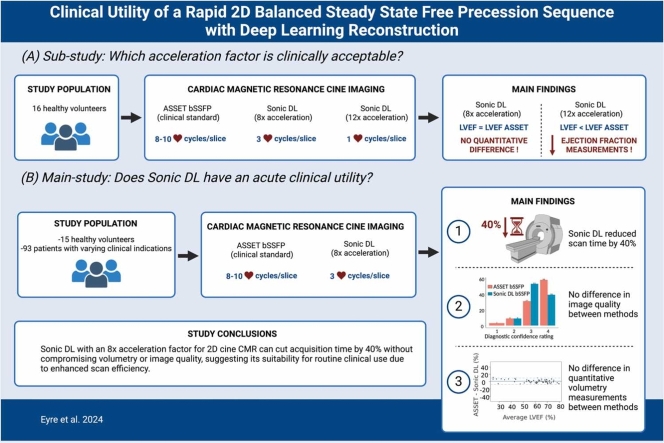

## 1 Introduction

Cardiovascular magnetic resonance (CMR) cine imaging is widely accepted as the gold-standard, non-invasive modality for visualization of cardiovascular anatomy and quantification of left-ventricular (LV) and right-ventricular (RV) function and volume measurements [1]. This is primarily due to its superior image quality (IQ) and high reproducibility compared to other imaging modalities [2]. The current method of choice to acquire these images is a breath-held balanced steady-state free precession sequence (bSSFP). This method allows for high temporal resolution and an optimal blood-pool-to-myocardium contrast [3]. Such contrast enhancement is crucial for clearly delineating cardiac structures, including trabecular tissue and the endocardial border. This reinforces the method’s highly reproducible and accurate capability for cardiac function and volume assessments [3].

While highly accurate, the current bSSFP method has several disadvantages. First, the method is lengthy. It requires patients to breath-hold (BH) for approximately 10–12 s—with intermediate periods of rest—for each of the two-dimensional (2D) slices required to achieve full heart coverage (11–12 short axis [SAx] slices and 3 long axis [LAx] slices) [3]. Second, BH-ing relies on patient compliance which may be compromised for several reasons. These reasons include anxiety, claustrophobia, age, respiratory complications, or other medical conditions [4]. This inconsistent BH-ing may result in misalignment of cardiac anatomy which may further complicate the future planning of slices, add scanning time, or challenge the clinical interpretation of images [5]. Finally, the bSSFP sequence is sensitive to magnetic field inhomogeneities. This sensitivity can lead to banding artifacts and/or signal loss near adjacent tissue areas with variations in magnetic susceptibility, such as the lung-heart interface or around metallic implants. These artifacts can potentially degrade the IQ of the heart structures and measurement accuracy in critical diagnostic regions.

In response to these challenges, advancements such as simultaneous multi-slice (SMS) acquisitions [6], [7], [8] and compressed sensing [9], [10], [11], [12], [13], [14] have emerged to shorten scan times.

SMS acquisitions enable the simultaneous capture of separate anatomical slices of the heart, increasing myocardial coverage in less time with fewer BHs [7]. When the optimal acceleration factor is employed, typically capturing three slices simultaneously, SMS acquisitions preserve signal-to-noise ratio (SNR) comparably to conventional bSSFP methods [7]. Although additional in-plane acceleration is achievable through varying coil sensitivities, cross-talk from simultaneous slice excitation presents a limitation [6]. Moreover, at higher magnetic fields, SMS acquisitions encounter constraints due to an increased specific absorption rate, further limiting the potential for acceleration [8].

Compressed sensing is another widely used technique to accelerate cine acquisitions. It exploits the inherent sparsity of CMR images in a transform domain to reconstruct images from fewer data points [12]. This method randomly under-samples magnetic resonance data to reduce structured artifacts through non-uniform sampling while maintaining essential structural image information. The reconstruction process is non-linear and iterative, which, while effective in reducing scan times, demands significant computational resources [12]. Compressed sensing may slightly reduce spatial resolution and risks missing the end-systolic phase. This could potentially underestimate end-systolic volume (ESV) and ejection fraction (EF) [12]. Thus, to achieve comparable IQ with reference methods without affecting quantitative volumetry measurements, conservative acceleration factors (2.5–3.5) are often used [10].

This study explores the clinical efficacy of Sonic DL, a GE Healthcare accelerated 2D bSSFP cine sequence. Sonic DL incorporates variable density k-t sampling [15] and deep learning (DL) reconstruction [16] for accelerated cine image acquisition. The variable density k-t undersampling is conducted over the phase encoding dimension. Different random shifts of phase encoding are added to different cardiac phases to create an incoherent sampling scheme across cardiac phase dimensions [16]. In the reconstruction, an unrolled convolutional neural network (CNN) architecture consisting of 12 unrolls is used. High IQ and fidelity are ensured by including a data consistency term and a CNN-based regularization term in each unroll [16]. The data consistency term uses coil sensitivities with assumptions derived from training data sets (“learned priors”) to inform image reconstruction from highly undersampled 2D data [16].

Each training dataset is acquired using a retrospectively triggered bSSFP cine sequence without any acceleration and using the Sonic DL sequence with variable density k-t undersampling. The undersampled cine data are used as training input, while the fully sampled cine data are used as the training label.

In preliminary studies, this approach was tested in a pediatric population [17] and in a small cohort of adult patients [18]. In the pediatric population, the Sonic DL sequence was significantly faster than the standard bSSFP sequence (0.9 min vs 3.0 min; p < 0.001) [17]. The IQ was only minimally lower for Sonic DL (3.8 ± 0.6) than for bSSFP (4.3 ± 0.6; p < 0.001) [17]. In the adult population, measurements of LV and RV volumes showed good agreement with standard images (p > 0.05) (r ≥ 0.76). However, LV mass (LVM) was underestimated in the Sonic DL images (109.8 ± 34.6 g) compared to bSSFP (116.2 ± 40.2 g; p = 0.0291) [18]. The authors found that the IQ scores of endocardial edge definition and motion artifacts were significantly impaired in the Sonic DL images. They attributed the difference in LVM to the technical limitations of the accelerated sequence [18]. The impact of using DL-processed undersampled images on diagnostic decision-making was not studied.

In this study, we aimed to 1) determine the optimal acceleration factor of Sonic DL using a small cohort of volunteers, and 2) assess IQ, accuracy, and diagnostic confidence of LV and RV volume and mass as measured in 2D Sonic DL images acquired using this optimal acceleration factor compared to a standard array coil spatial sensitivity encoding (ASSET) bSSFP sequence in a larger cohort of adult patients with various cardiac diseases.

## 2 Methods

### 2.1 Study population

For our sub-study, 16 healthy volunteers were prospectively recruited. Each participant gave written informed consent before their CMR scan. Participants were ineligible for recruitment if they had contraindications to CMR, including claustrophobia, pregnancy, non-compatible pacemaker/defibrillator devices, or intraocular/intracranial metallic materials. Volunteers were considered healthy if they were non-smoking, had a body mass index (BMI) of less than 30, were not taking any medications, and had no significant past medical history.

For our larger study, 93 patients with clinical indications for a CMR exam (72 [67%] men, age 53.3 ± 15.3 years) and 15 healthy volunteers were prospectively recruited. All subjects gave written informed consent. Ineligibility and healthy volunteer recruitment followed the same conditions as in our sub-study. Patients with the following clinical indication were enrolled: atrial fibrillation (n = 38), suspected myocarditis (n = 23), suspected or known coronary artery disease (n = 13), hypertrophic cardiomyopathy (n = 9), and other non-ischemic cardiomyopathies (n = 10).

### 2.2 CMR protocol

CMR images were acquired with a clinical MRI system (Premier™ 3T, GE Healthcare, Milwaukee, Wisconsin, USA) using high-channel-count phased-array coils, AIR™ surface coil (30 anterior coil channels + 60 posterior coil channels). For patients, the scans were performed according to indication-specific protocols (Supplementary Table 1). In our sub-study, all participants underwent a conventional 2D array coil spatial sensitivity encoding (ASSET) bSSFP sequence and the proposed 2D Sonic DL bSSFP cine sequences at two acceleration factors (8× and 12×). These were performed using identical positioning and orientation. In our larger study, both, a conventional 2D ASSET bSSFP sequence and the 2D Sonic DL sequence at an acceleration factor of 8× were performed using identical positioning and orientation. Images were acquired in two-chamber (2Ch), three-chamber (3Ch), four-chamber (4Ch) views, and as a SAx stack through both ventricles (11–12 slices) at end-expiration during several BHs. In cases where susceptibility artifacts at the lung-myocardium interface were present, frequency scouting was used. This involved conducting a series of preliminary scans at different frequency offsets. The setting that best reduced this artifact was selected and applied to the bSSFP sequence.

The 2D ASSET bSSFP cine with one BH per slice was acquired using the following imaging parameters: in-plane resolution 1.8 mm × 1.8 mm; slice thickness 8 mm; repetition time/echo time (TR/TE) 3.1 ms/1.2 ms; flip angle 55°; bandwidth 125 Hz; slice gap 2 mm; acceleration factor 2. The 2D Sonic DL bSSFP cine sequence allowed for the acquisition of 3–5 slices per BH, using the following imaging parameters: in-plane resolution 1.8 mm × 1.8 mm; slice thickness 8 mm; TR/TE 2.9 ms/1.1 ms; flip angle 49°; bandwidth 125 Hz; slice gap 2 mm; acceleration factor 8 or 12. The Sonic DL bSSFP cine data were reconstructed inline with an unrolled (combining iterative and DL techniques) neural network reconstruction prototype [16]. This prototype included a data consistency update and a CNN-based regularization term on spatiotemporal-split convolutions. The network was trained on 6480 fully sampled 2D bSSFP cine images for approximately 3 days on an NVIDIA V100 graphics processing unit (GPU). The average inline reconstruction time for the entire Sonic DL acquisition was 340 s. The acquisition time was recorded for each of the two cine sequences in the larger study.

### 2.3 Image analysis

All CMR images were analyzed offline by two blinded readers at a core lab (McGill University Health Centre, Montreal, Quebec, Canada) using commercial software (cvi42™, Circle Cardiovascular Imaging Inc., Calgary, Alberta, Canada). The cine LAx and SAx views were used for quantitative LV/RV functional and volumetric measurements. Endocardial contours were semi-automatically traced using the built-in “threshold tool.” This tool separates the high signal-intensity blood-pool pixels from the lower-intensity myocardial pixels. Often, manual adjustments were used with particular attention to anatomical details to ensure that the trabecular tissue and papillary muscles were excluded from the blood-pool area [1]. In contrast, epicardial contours were manually traced. Both these contours were drawn at end-diastole and end-systole. In basal slices, contours were carefully drawn to include the LV outflow tract into the LV volume up to the level of the aortic valve cusps. LV and RV volumes at end-diastole and end-systole, EF, and LVM, including their respective indexes normalized to the body surface area (BSA) [19], and height, were calculated using the Simpson method for the SAx stack, and a biplane method for the LAx views [20]. End-diastolic and end-systolic phases were defined by the largest and smallest area measured in a mid-ventricular slice, respectively [21]. LVM was measured in the end-systolic phase to reduce the effect of partial volume effects in trabecular layers [22]. In 24 patients, we also measured global peak radial and circumferential strain using the feature tracking method as previously described [23].

The IQ between the two techniques was compared using both qualitative and quantitative metrics.

The qualitative IQ assessment was performed in LAx and SAx views. Two blinded clinical readers (one radiologist with 9 years of experience reading clinical CMR and one cardiologist with 6 years of experience reading clinical CMR) were asked to rate IQ regarding their diagnostic confidence using a 4-point ordinal scale: 1: no diagnostic confidence (non-interpretable); 2: low diagnostic confidence (poor IQ, significant artifacts); 3: moderate diagnostic confidence (good overall IQ with one or two views with poorer IQ); 4: high diagnostic confidence (high IQ, no views with significantly impaired IQ).

For the quantitative IQ assessment, the contrast difference between the blood pool and the myocardium, as well as endocardial border sharpness, was evaluated in a mid-ventricular SAx slice at end-diastole. The contrast difference was calculated as the difference in the average myocardium signal intensity from the average blood-pool signal intensity:

Contrast difference=Average blood pool signal intensity−Average myocardial signal intensity.

**Fig. 1:**
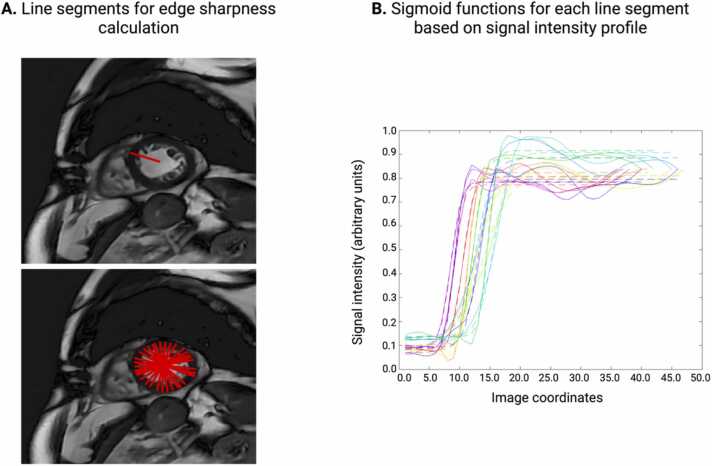
Schematic depiction of the calculation of endocardial edge sharpness measurements. (A) Radial lines drawn in an orthogonal fashion from the center of the LV cavity to the subepicardial myocardial boundary to compute a signal intensity profile. (B) Endocardial edge sharpness was calculated by taking the average slope of the sigmoid functions that were fit to the signal intensity profile. *LV* left ventricle.

To measure endocardial edge sharpness, a three-step procedure was used. First, masks for blood pool and myocardium were generated manually using MatLab R2021b (MathWorks, Natick, Massachusetts, USA). Then, several line segments were drawn orthogonal to the blood pool and myocardial boundary, allowing for a signal intensity profile to be computed (Fig. 1). Edge sharpness was calculated by taking the average slope of the sigmoid functions that were fit to the signal intensity profile of the line segment (Fig. 1). For this assessment, we chose to evaluate a mid-ventricular slice below the level of the papillary muscles so that the papillary muscles would not interfere with our calculations. All subjects had the same number of orthogonal lines computed for analysis.

### 2.4 Inter-observer quality assurance

The evaluation of inter-observer reliability was performed for LV and RV volumetry, and LVM by certified core lab readers. Eighty randomly selected CMR studies were used. Inter-class correlations (ICC) and bias in measurements were used to assess the interobserver variability [24].

### 2.5 Statistical analysis

Continuous variables were presented as means with standard deviations (SD) or as medians with interquartile range, while categorical variables were presented as numbers or percentages. Normality was verified using the Shapiro-Wilk test. Differences between means were evaluated using paired student t-tests for parametric data or the Mann-Whitney test, or Wilcoxon signed-rank tests for non-parametric data. A repeated measures analysis of variance (ANOVA) was used to compare LV volumetry, function, and mass measurements between ASSET and Sonic DL at the two different acceleration factors. A Bland-Altman analysis was performed to compare LV and RV volumetric and LVM measurements between 2D ASSET bSSFP and 2D Sonic DL bSSFP cine methods. Correlation between parameters was also assessed using Pearson’s correlation analysis. Statistical significance was set at p < 0.05. All statistical analyses were performed using R (version 3.6.3. R Foundation for Statistical Computing, Vienna, Austria).

## 3 Results

### 3.1 Sub-study

**Fig. 2:**
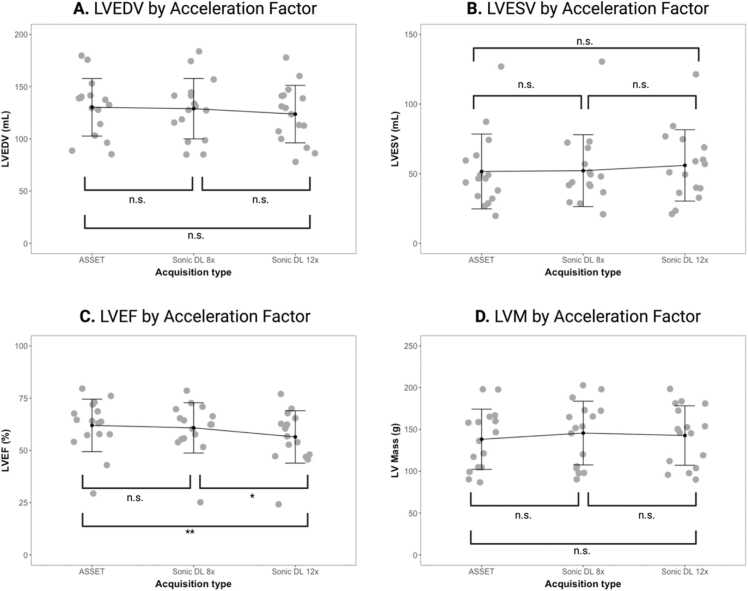
Comparison of left-ventricular (LV): (A) end-diastolic volume (EDV), (B) end-systolic volume (ESV), (C) ejection fraction (EF) and (D) mass between ASSET and Sonic DL at an acceleration factor of 8 and 12. A repeated measures ANOVA was used to compare means between methods. A p-value of <0.05 was considered statistically significant. *ASSET* array spatial sensitivity encoding technique, *DL* deep learning, *n.s.* non-significant, *LVM* left ventricular mass, *ANOVA* analysis of variance.

The cine CMR protocol was successfully performed in all 16 subjects. No significant differences were found between the three methods in LV end-diastolic volume (LVEDV), LVESV, nor LVM measurements (Fig. 2). However, Sonic DL with an acceleration factor of 12 reduced the LVEF measurements by 6% (5.51/90.16) compared to our standard ASSET method (p = 0.004) and by 4% (4.32/88.98) compared to Sonic DL with an acceleration factor of 8 (p = 0.015). LVEF measurements by Sonic DL with an acceleration factor of 8 showed no significant differences compared to ASSET.

### 3.2 Population

Between June 2020 and June 2022, 93 patients (72 [67%] men, age 53.3 ± 15.3 years) with clinical indications for a CMR and 15 healthy volunteers were prospectively recruited. The protocol was successfully performed in all subjects. Details of the baseline demographics and clinical CMR indications for the participants recruited are presented in Table 1.

**Table 1:** Baseline demographics and clinical indications for subjects enrolled in this study (n = 108).

*Baseline variables*	*Mean*	*SD*
Age (years)	53	15.3
Body weight (kg)	80.7	17.7
Height (cm)	173.5	10.3
BSA (m^2^)	1.96	0.26
BMI (kg/m^2^)	26.23	4.46
Systolic blood pressure (mmHg)	124.77	16.96
Diastolic blood pressure (mmHg)	74.58	11.85
Heart rate (bpm)	71.24	15.81
		
*Clinical indications for CMR*	*Number*	*Percentage*
Hypertrophic cardiomyopathy	9	8
Atrial fibrillation	38	35
Coronary artery disease	13	12
Myocarditis/pericarditis/inflammation	23	21
Other NICMP	10	10
Healthy volunteer	15	14

*SD* standard deviation*, BSA* body surface area*, BMI* body mass index*, CMR* cardiovascular magnetic resonance*, NICMP* non-ischemic cardiomyopathy*.*

### 3.3 Comparison in scan time and image quality

**Fig. 3:**
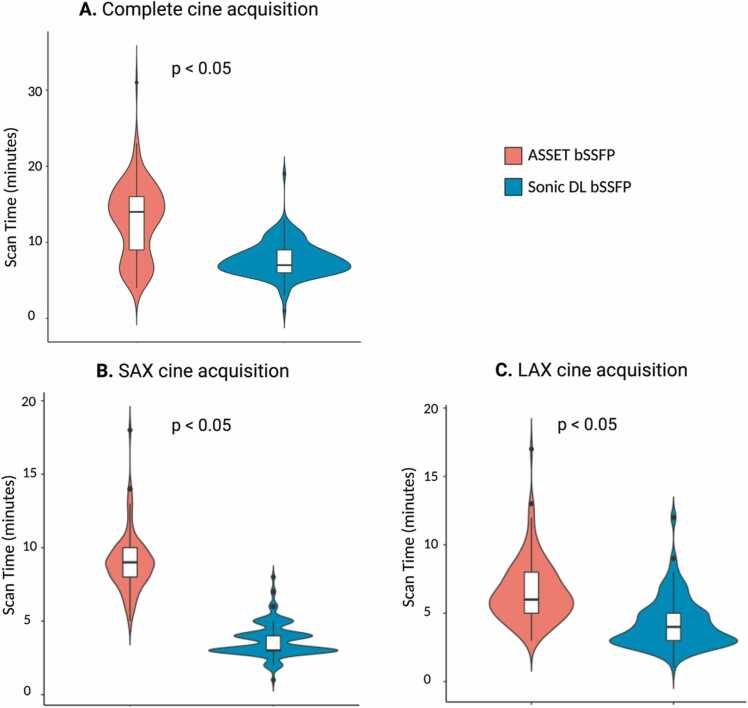
Comparison of scan time between methods. (A) Comparison of scan time between acquisition methods of the complete short-axis (SAx) and long-axis (LAx) views. (B) Comparison of scan time between methods of the SAx stack. (C) Comparison of scan time between methods of the three LAx views. *ASSET* array coil spatial sensitivity encoding, *bSSFP* balanced steady-state free precession, *DL* deep learning.

The Sonic DL bSSFP cine sequence significantly reduced the acquisition time of cine images when compared to ASSET bSSFP (37% (5.98/16) decrease in total acquisition time, p < 0.0001) (Fig. 3). The acquisition time reduction was particularly evident for the SAx stack (mean acquisition time of SAx using Sonic DL bSSFP: 3.66 ± 1.1 min vs ASSET bSSFP: 8.95 ± 1.97 min). The acquisition of 2Ch, 3Ch, and 4Ch LAx images was also significantly shorter when using Sonic DL bSSFP cine (mean acquisition time of 3 LAx views using Sonic DL bSSFP: 4.26 ± 1.86 min vs ASSET bSSFP: 6.56 ± 2.23 min).

**Fig. 4:**
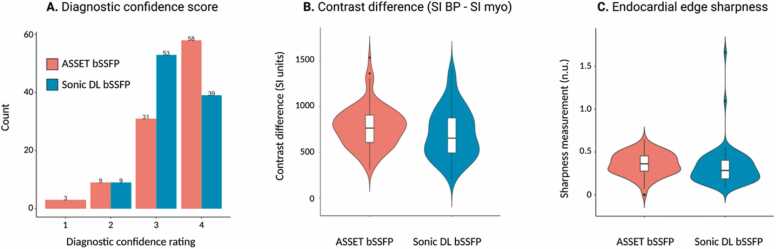
Results from image quality assessment. (A) Diagnostic confidence scores were obtained from two experienced clinical readers. Images were anonymized to sequence type and randomized with respect to the order they were presented to the readers. 1: No diagnostic confidence (not interpretable); 2: low diagnostic confidence (poor image quality); 3: medium diagnostic confidence (good overall image quality with one or two views with poorer IQ); 4: high diagnostic confidence (perfect image quality). (B) Contrast difference measurements were taken between the blood pool and the myocardium by subtracting the signal intensity of the myocardium from that of the blood pool. (C) Endocardial edge sharpness was calculated by taking the average slope of the sigmoid functions that were fit to the signal intensity profile of the line segment than was drawn orthogonal to the myocardial and blood pool border. *ASSET* array coil spatial sensitivity encoding technique, *bSSFP* balanced steady-state free precession, *DL* deep learning, *SI* signal intensity, *BP* blood pool, *Myo* myocardium, *IQ* image quality.

**Fig. 5:**
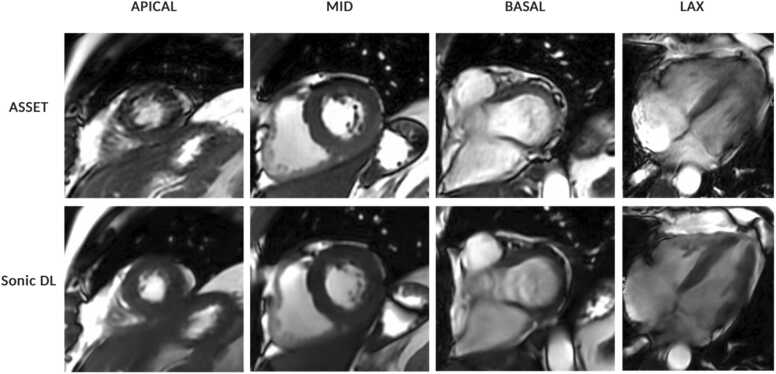
A representative set of cine images at end-systole and end-diastole from a patient with atrial fibrillation. *ASSET* array coil spatial sensitivity encoding technique, *bSSFP* balanced steady-state free precession, *DL* deep learning, *LAx* long axis.

Quantitative and qualitative IQ were similar between methods (p > 0.05) (Table 2, Fig. 4). A greater number of standard ASSET cases were rated with a high diagnostic confidence score compared to Sonic DL (58 vs 39 cases) (Fig. 4). However, the ASSET method contained three cases that were rated as non-interpretable, while Sonic DL had no such cases (Fig. 4). Fig. 5 shows representative cine images using both methods in a patient with atrial fibrillation.

**Table 2:** Image quality assessment of short-axis images acquired with the ASSET bSSFP or the Sonic DL bSSFP sequence.

Parameters	ASSET bSSFP	Sonic DL bSSFP	p-value
User-rated image quality	3.4 ± 0.8	3.3 ± 0.6	0.197
Contrast difference (SI BP − SI myocardium)	777 ± 225	707 ± 274	0.049
Blood pool to myocardial edge sharpness	0.36 ± 0.13	0.32 ± 0.22	0.241

User-rated image quality was conducted using a 4-point ordinal scale by 2 experienced clinical readers, where 5 represented images with the best image quality. Contrast difference was measured between the blood pool and myocardium by taking the difference in myocardium signal intensity from the signal intensity of the blood pool. The blood pool to myocardial edge sharpness was calculated as the mean fitted slope of a sigmoid to the signal intensity profile of pixels in the blood pool as they transitioned to the myocardium. Paired statistical tests were used to compare means between the scores of the two methods. A p-value of <0.05 was considered statistically significant. Values are presented as means ± standard deviation.

*ASSET* array spatial sensitivity encoding technique*, bSSFP* balanced steady-state free precession*, DL* deep learning*, SI* signal intensity*, BP* blood pool*.*

### 3.4 Comparison of quantitative measurements

**Fig. 6:**
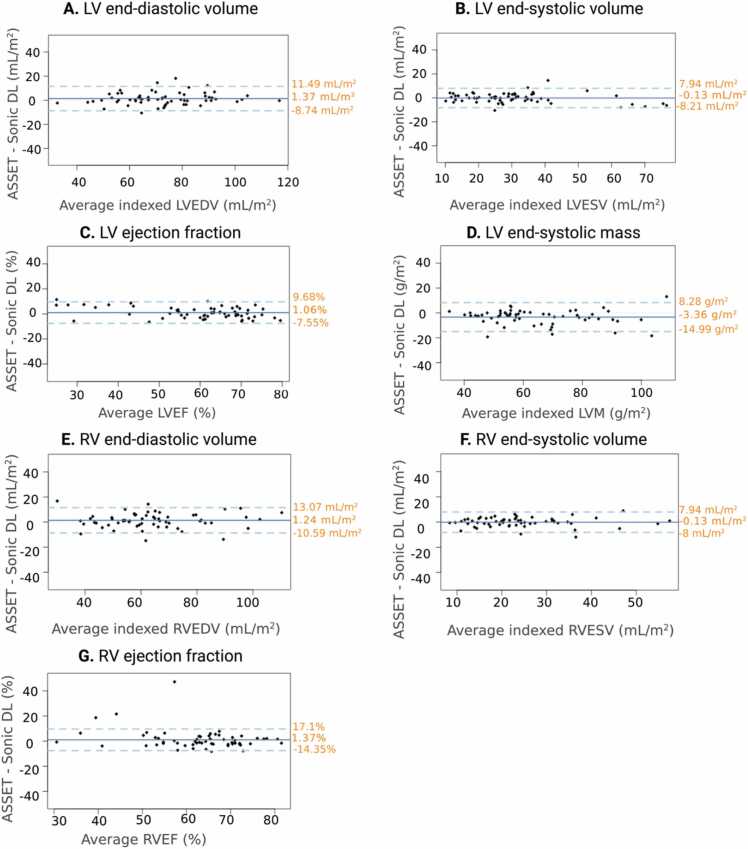
Bland-Altman plots displaying the similarity of measured ventricular function metrics using ASSET bSSFP and Sonic DL bSSFP sequence. The solid blue line represents the average bias between measurements. The dotted lines represent the upper and lower 95% limits of agreement. Bland-Altman plots displayed for (A) LVEDV, (B) LVESV, (C) LVEF, (D) LVM, (E) RVEDV, (F) RVESV, (G) RVEF. LV: left ventricle, *ASSET* array coil spatial sensitivity encoding technique encoding, *DL* deep learning, *RV* right ventricle, *EDV* end-diastolic volume, *ESV* end-systolic volume, *EF* ejection fraction, *LVM* left ventricular mass.

**Fig. 7:**
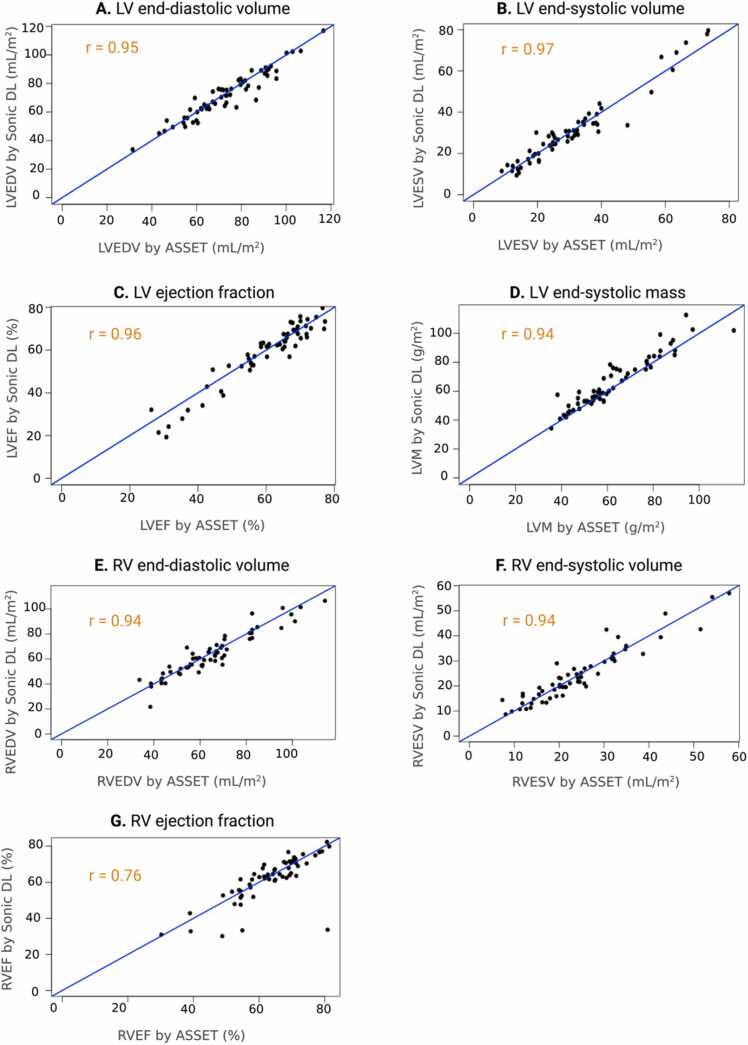
Scatter plot displaying the relationship between measured ventricular function metrics by ASSET bSSFP and Sonic DL bSSFP. Scatter plots are displayed for (A) LVEDV, (B) LVESV, (C) LVEF, (D) LVM, (E) RVEDV, (F) RVESV, and (G) RVEF. *LV* left ventricle, *ASSET* array coil spatial sensitivity encoding technique, *DL* deep learning, *RV* right ventricle, *EDV* end-diastolic volume, *ESV* end-systolic volume, *EF* ejection fraction, *LVM* left ventricular mass, *r* correlation coefficient.

BSA-indexed-LV ESV, LVEF, indexed-RV end-diastolic volume (EDV), indexed-RVESV, and RVEF, calculated from the SAx stack, were statistically not different between Sonic DL and ASSET bSSFP (p > 0.05) (Table 3). However, Sonic DL was found to under-estimate indexed-LVEDV (Sonic DL: 71.95 ± 16 mL/m^2^, ASSET: 73.32 ± 17 mL/m^2^, p = 0.0437) and over-estimate LVM (Sonic DL: 66.29 ± 17 g/m^2^, ASSET: 62.77 ± 17 g/m^2^, p < 0.0001) compared to ASSET bSSFP (Table 3). The average difference between methods for each parameter was 1.37 ± 5.2 mL/m^2^, −0.13 ± 4.1 mL/m^2^, 1.06 ± 4.4%, −3.36 ± 5.9 g/m^2^, 1.24 ± 6 mL/m^2^, −0.04 ± 3.6 mL/m^2^, and 1.37 ± 8.02% for indexed-LVEDV, indexed-LVESV, LVEF, indexed-LVM, indexed-RVEDV, indexed-RVESV, and RVEF, respectively (Table 3, Fig. 6). We found no differences in the comparison between methods when patients were stratified by disease or healthy volunteers (Supplementary Table 2, Supplementary Fig. 1). Measured volumetry and LVM values indexed to height are found in Supplementary Table 3. All functional parameters demonstrated a strong correlation (r > 0.7) between cine methods (Table 3, Fig. 7).

**Table 3:** Comparison of measured functional parameters between ASSET bSSFP cine and Sonic DL bSSFP cine using SAx stack images.

Parameters	LVEDV (mL/m^2^)	LVESV (mL/m^2^)	LVEF (%)	LVM (g/m^2^)	RVEDV (mL/m^2^)	RVESV (mL/m^2^)	RVEF (%)
ASSET bSSFP	73.32 ± 17	30.57 ± 15	59.98 ± 13	62.77 ± 17	64.79 ± 18	24.16 ± 11	63.47 ± 10
Sonic DL bSSFP	71.95 ± 16	30.7 ± 16	59.04 ± 15	66.29 ± 17	63.74 ± 17	24.14 ± 11	62.26 ± 12
ASSET − Sonic DL	1.37 ± 5.2	−0.13 ± 4.1	1.06 ± 4.4	−3.36 ± 5.9	1.24 ± 6	−0.04 ± 3.6	1.37 ± 8.02
p-value	0.0437	0.8006	0.06818	< 0.0001	0.1199	0.9352	0.1936
r	0.952	0.968	0.958	0.942	0.941	0.943	0.764

All volumes were indexed to body surface area. Statistical significance was considered when p < 0.05. Values are presented as means ± standard deviation.

*ASSET* array coil spatial sensitivity encoding technique*, bSSFP* balanced steady-state free precession*, DL* deep learning*, SAx* short axis*, LVEDV* left ventricular end-diastolic volume*, LVESV* left ventricular end-systolic volume*, LVEF* left ventricular ejection fraction*, LVM* left ventricular mass*, RVEDV* right ventricular end-diastolic volume*, RVESV* right ventricular end-systolic volume*, RVEF* right ventricular ejection fraction*, r* correlation coefficient*.*

For strain measurements, global peak radial and circumferential strain measurements were highly correlated (r > 0.9) and were statistically not different (p > 0.05) between both cine methods (Table 4).

**Table 4:** Comparison of measured strain values between ASSET bSSFP cine and Sonic DL bSSFP cine.

Parameters	Global peak radial strain (%)	Global peak circumferential strain (%)	Global peak longitudinal strain (%)
ASSET bSSFP	31.6 ± 9.4	−17.4 ± 3.6	−14.0 ± 3.2
Sonic DL bSSFP	31.4 ± 8.4	−17.3 ± 3.2	−12.1 ± 4.0
ASSET − Sonic DL	0.21 ± 3.7	−0.15 ± 1.3	−1.97 ± 2.6
p-value	0.78	0.60	0.001
r	0.92	0.93	0.66

Radial and circumferential strain were calculated using SAx views, while longitudinal strain was calculated using LAx views. Statistical significance was considered when p < 0.05. Values are presented as means ± standard deviation.

*ASSET* array coil spatial sensitivity encoding technique*, bSSFP* balanced steady-state free precession*, DL* deep learning*, LAx* long axis*, SAx* short axis*, r* correlation coefficient*.*

### 3.5 Inter-observer reliability

A summary of the inter-observer reliability results is listed in Table 5. Moderate to excellent inter-observer variability was demonstrated for all measured parameters. Measurements between observers tended to differ slightly more with the Sonic DL bSSFP sequence compared to ASSET, and when measuring right ventricular volumes.

**Table 5:** Inter-observer variability of ASSET and Sonic DL measurements.

	LVEDV (mL)	LVESV (mL)		LVEF (%)		LVM (g)	RVEDV (mL)	RVESV (mL)	RVEF (%)
ASSET (observer 1)	136.6 ± 37	56.1 ± 33		60.9 ± 15		129.7 ± 40	145.5 ± 42	65.9 ± 28	55.4 ± 11
ASSET (observer 2)	136.3 ± 37	56.5 ± 28		60.1 ± 13		118.6 ± 39	125.6 ± 42	50.1 ± 24	60.8 ± 10
ICC	0.94	0.91		0.91		0.91	0.87	0.786	0.702
CI 95% lower/upper	0.88/0.97	0.83/0.96		0.82/0.96		0.61/0.97	−0.02/0.97	−0.04/0.94	0.26/0.87
Bias ± variability	1.8 ± 5.4	3.0 ± 6.1		1.5 ± 3.2		11.1 ± 13.3	19.8 ± 10.2	15.8 ± 9.8	5.4 ± 7.0
Sonic DL (observer 1)	134.8 ± 37	57.0 ± 37		60.0 ± 17		137.1 ± 42	146.0 ± 43	69.0 ± 33	53.6 ± 13
Sonic DL (observer 2)	134.9 ± 35	57.8 ± 30		58.6 ± 15		122.7 ± 39	122.0 ± 38	48.8 ± 24	60.6 ± 12
ICC	0.94	0.89		0.92		0.85	0.83	0.72	0.8
CI 95% lower/upper	0.87/097	0.78/0.95		0.83/0.96		0.49/0.94	−0.04/0.96	−0.06/0.92	0.04/0.94
Bias ± variability	0.06 ± 13.4	0.8 ± 15.8		1.4 ± 6.5		14.4 ± 18.4	24.0 ± 10.1	20.2 ± 13.1	7.0 ± 5.4

Values are presented as means ± standard deviation.

*ASSET* array coil spatial sensitivity encoding technique*, DL* deep learning*, ICC* intraclass correlation coefficient*, LVEDV* left ventricle end-diastolic volume*, LVESV* left ventricle end-systolic volume*, LVEF* left ventricle ejection fraction*, LVM* left ventricle mass*, RVEDV* right ventricle end-diastolic volume*, RVESV* right ventricle end-systolic volume*, RVEF* right ventricle ejection fraction*, CI* confidence interval*.*

## 4 Discussion

Our results indicate that an accelerated 2D cine method with DL reconstruction may reduce CMR cine acquisition time. This method does not significantly affect volumetry or IQ and does not compromise diagnostic confidence. These results suggest that Sonic DL may have a direct clinical application for CMR cine imaging.

CMR is generally perceived as a high-cost investigational tool [25], limited by long exam times. These lengthy exam times limit the efficiency of clinical CMR, often resulting in reduced access to scanners and long waiting lists [26]. Since cine sequences are an essential component of CMR evaluation [3], their lengthy acquisition contributes to this problem. ASSET bSSFP as a clinical standard method for obtaining CMR cine data requires multiple sequential BHs to acquire 14–15 different 2D views, encompassing both SAx and LAx views of the heart [3]. Effective acquisition relies on patient cooperation for BH-ing and accurate electrocardiogram (ECG) signal capture to synchronize with the heart rhythm. However, patients requiring cardiac MRI often present with conditions that challenge their BH-ing capacity, exhibit high heart rate variability, or suffer from severe arrhythmias. These factors complicate image acquisition and extend the time needed to comprehensively image the heart.

While free-breathing, three-dimensional (3D), SMS, or other highly accelerated acquisitions [6], [7], [8], [27], [28], [29], may address issues related to BH-ing, anatomical 2D slice misalignment, or even ECG-triggering, they are not yet widely adopted in clinical settings due to their own set of complications. Free-breathing techniques, though alleviating BH issues, may not necessarily shorten acquisition times and may require patients to remain immobile for extended periods. SMS methods may offer further scan efficiency compared to Sonic DL by capturing multiple slices simultaneously. However, they introduce the potential for slice cross-talk, which may negatively affect IQ and slice alignment [7]. Even if the acquisition is accelerated, the often significant computational demands for data reconstruction, potentially extending over hours or even days, delay clinical decision-making for patients. In these cases, if IQ is compromised, this may even necessitate a repeat exam further reducing clinical efficiency. Additionally, the reconstruction algorithms used may oversimplify cardiac dynamics or require tedious manual tuning of regularization parameters [27], [30], limiting their practicality compared to the established efficiency and reliability of 2D cartesian cine methods.

The 2D Sonic DL sequence used in this study may not achieve the same level of time efficiency as other accelerated methods [31], [32]. However, it may offer a preferable balance between speed and IQ without introducing lengthy reconstruction times in the clinical setting. In our study, scan time was reduced by 40% compared to ASSET (Fig. 3) with an average reconstruction time of 5–6 min for the entire imaging series. This acceleration offers patients a shorter BH time per slice or fewer BH’s overall for the complete cine acquisition. In addition, image reconstruction is complete before the patients' examination is finished, reducing the potential for repeat examinations due to poor IQ. Further, this study employed a prototype reconstruction implemented on a CPU. With further model optimization and GPU implementation, we anticipate that reconstruction times could be reduced to mere seconds. This improvement could imply that cine images are reconstructed before the next series is acquired, meeting current clinical demands efficiently.

The acceleration offered by Sonic DL is achieved by leveraging a Cartesian variable density k-t-space sampling pattern [15]. This distinguishes it from ASSET’s uniform under-sampling approach. By focusing on denser sampling in the center of k-t space—where the raw data contributing to image contrast and structure definition reside [34]—Sonic DL minimizes the loss of important image information, with under-sampling artifacts manifesting as noise [15]. This strategy, coupled with sophisticated reconstruction techniques, can preserve the diagnostic integrity of images. Conversely, applying an equivalent acceleration factor to ASSET might lead to fold-over artifacts, challenging to eliminate even with multi-channel receiver coil technology.

To recover the missing data from under-sampling, Sonic DL uses a data-driven unrolled CNN reconstruction [11], [16] and mirroring techniques such as CINENet [35]. The data-driven approach uses information from coil sensitivities and “learned priors” from network training to balance data consistency with network regularization [16]. The incorporation of DL into the reconstruction facilitates faster reconstruction times and allows further image acceleration as DL may learn better priors for image recovery [16], [35], [36], [37], [38], [39], [40].

While DL may indeed learn optimal regularization weights and imaging priors for image recovery, our sub-study found that Sonic DL with an acceleration factor of 12 underestimated LV volumetry and LVEF measurements. This suggests that compression of cardiac motion may occur with Sonic DL at higher acceleration factors. This issue is common with regularized reconstruction methods [9], [16], [33]. Over-regularization, used to enforce sparsity, can smooth out important anatomical details, leading to a blurred appearance in the reconstructed image. Our findings indicate that while Sonic DL at 8× acceleration is effective, further future investigation into the issue of temporal blurring may be valuable to optimize this imaging sequence and reconstruction algorithm, potentially allowing for even higher acceleration rates to be achieved.

Techniques, such as CINENet, support 3D acquisition through an additional phase encoding dimension, offering greater acceleration potential, yet 2D cine remains the clinical preference. This is due to shorter BH requirements and superior blood-pool-to-myocardium contrast. Despite needing BHs, Sonic DL reduces the overall number of BHs needed to achieve full anatomical coverage. This lowers the risk of slice misalignment due to inconsistent BH-ing and reduces the need for repetitions. This technique may also be easier for patients who struggle to adhere to the breathing maneuvers.

From a clinical perspective, this study found that Sonic DL’s did not impair the diagnostic integrity of images, as rated by two expert clinical readers. Even though more ASSET cases were rated as having near-perfect IQ by clinical readers (Fig. 3), all Sonic DL cases were rated as diagnostic. This was not the case for ASSET, suggesting that Sonic DL, while not improving subjective IQ under perfect imaging conditions, appears to improve IQ under suboptimal imaging conditions. This is especially important in high-throughput settings, where time restrictions often do not allow for individually optimizing scanner settings.

Clinicians require the diagnostic integrity of images to remain uncompromised. This can be determined through their satisfaction with images as well as through specific quantitative metrics of IQ. Precise quantification of blood volumes and mass necessitates accurate demarcation of the endocardial and epicardial borders, assessable by edge sharpness or contrast differentiation metrics. The use of regularization terms in Sonic DL's reconstruction process, aimed at noise reduction, renders direct comparisons of contrast-to-noise ratio (CNR) or SNR infeasible in this study. Instead, we evaluated the contrast differences between tissues by measuring the signal intensity variance between the myocardium and the blood pool as endocardial edge sharpness or contrast difference. We chose to evaluate a mid-ventricular slice below the level of the papillary muscles to avoid interference of papillary muscles in any calculations. Our study observed no significant discrepancies in endocardial edge sharpness or myocardium-to-blood-pool contrast between ASSET and Sonic DL. These conclusions were corroborated by quantitative volumetry and functional assessments, which also showed no statistically significant differences between the methods.

Although LV and RV volumetry measurements were similar between ASSET and Sonic DL, our study found that LVM was overestimated using Sonic DL. Given LVM's association with severe cardiovascular conditions and its significance in diagnosing hypertrophic cardiomyopathies and other infiltrative diseases, accuracy in its measurement is crucial [41], [42]. Despite observing a statistically significant difference, the magnitude of this variance (∼3 g/m^2^) is unlikely to misclassify a patient's myocardial mass as normal or abnormal. Although the variation in LVM measured may not have clinical relevance, it suggests caution when using Sonic DL in scenarios where precise mass measurements are critical [3].

Our findings with LVM measurements are in contrast to previous results which found that LVM was under-estimated by sonic DL [18]. The difference in findings may be attributed to a difference in contouring methods, as the previous study excluded trabeculations and papillary muscles from myocardial mass while this study included them [18].

## 5 Limitations

This study must be interpreted in the context of its limitations. This study was conducted at a single center with a medium sample size. These results have yet to be replicated in larger cohorts. We studied a population that may not be representative of those in other centers, and results may not be generalizable to pathologies not included here, such as diseases with complex anatomy (i.e. congenital) or thin myocardial walls (i.e. excessive LV trabeculation). ASSET bSSFP cine is still prone to measurement errors so the lack of a third measurement tool as a reference limits the interpretability of the results. Quantitative IQ was only measured in one mid-ventricular SAx slice at end-diastole, arguably the imaging area with fewer problems. Therefore, these results may not accurately depict IQ for more problematic regions of the heart such as the apex with its increased amount of trabeculations, or at the basal region. Finally, only the border between the blood pool and myocardium was measured in terms of its border sharpness and CNR. Of note, we found that Sonic DL may overestimate LVM without affecting volumetry. Thus, the difference may be due to an altered visual appearance of epicardial borders in Sonic DL images. This should be further investigated.

In this study, Sonic DL was unable to reach acceleration rates as high as similar methods cited in literature [31], [32]. The reasons for this may be explained by an introduction of temporal blurring at higher acceleration rates. The spatiotemporal-split convolutions used in the reconstruction framework, while useful for managing the complexity of dynamic imaging data, can contribute to temporal blurring if they inadequately capture temporal dependencies or overly prioritize spatial features. While this potential issue did not prevent Sonic DL from ultimately reducing scan time without affecting quantitative LV volumetry or IQ, it suggests that the framework may be further improved with a more thorough investigation into this topic.

Similarly, we focused on exploring the performance of Sonic DL at a specific acceleration factor of 8. Notably, previous research indicates that lower acceleration factors, such as 4, can yield results more closely aligned with standard cine sequences while still offering the benefits of accelerated acquisitions [43]. The choice of acceleration factor is not merely a technical consideration but also a clinical one, as it may provide varied advantages depending on the disease context. For conditions requiring precise volumetry, function, and mass measurements, lower acceleration factors are preferable to ensure accuracy. Conversely, in scenarios where patients face challenges with arrhythmia or limited BH capacity, and where precise measurements are less critical for diagnosis, higher acceleration factors could be more suitable. This differentiation underscores the need to tailor the acceleration factor based on specific diagnostic requirements, although this study did not extend to evaluating Sonic DL's generalizability across these different settings.

## 6 Conclusion

Undersampled k-space sampling methods combined with DL reconstruction can be considered an efficient tool to reduce CMR scan times [33]. This would increase the efficiency of CMR scanning without compromising its clinical utility. Shorter scan times may also improve the patient experience. If these results can be replicated in larger, multi-center trials, Sonic DL has the potential to replace traditional, slower imaging techniques in routine CMR imaging.

## Funding

This study received funding from MEDTEQ+ and GE Healthcare.

## Author contributions

K.E. was involved in patient and healthy volunteer recruitment, conceptualization of study analysis, performance of data and statistical analysis, and manuscript writing. M.J.R. performed all image analysis (contouring) and performed qualitative image quality scores. M.L. performed all CMR acquisition of participants and participated in image analysis (contouring). J.M. provided technical assistance throughout the study whenever needed and assisted with manuscript writing. E.H. contributed to the conceptualization of the study design and performed a pilot study using the research sequence investigated in this study. N.A. assisted with all data organization, anonymization, and randomization, and contributed to image analysis (contouring). J.P. contributed to the structuring and organization of the enclosed manuscript. M.A.J. contributed to the conceptualization of the study and is also the representative for GE Healthcare which provided funding for this study. X.Z. contributed to the novel sequence development and development of the reconstruction pipeline. M.G.F. contributed to the conceptualization of the study and reviewed all contouring to ensure accuracy. M.C. contributed to the conceptualization of the study analysis and performed qualitative image quality scores. All authors were asked to review the manuscripts’ content after it was drafted.

## Ethics approval and consent

This study was given ethics approval by the McGill University Health Centre (MUHC) for Applied Ethics (MUHC authorization number: CMR10/2020-6128).

## Consent for publication

Not applicable.

## Declaration of competing interests

The authors declare the following financial interests/personal relationships which may be considered as potential competing interests: Matthias Friedrich reports financial support was provided by MEDTEQ and GE Healthcare. Matthias Friedrich reports a relationship with Circle Cardiovascular Imaging Inc that includes board membership. Martin Janich, Junjie Ma, and Xucheng Zhu report a relationship with GE Healthcare that includes employment. The other authors declare that they have no known competing financial interests or personal relationships that could have appeared to influence the work reported in this paper.

## Data Availability

The datasets used and/or analyzed during the current study are available from the corresponding author on reasonable request.
