# Supplementary material for: Clinical utility of a rapid two-dimensional balanced steady-state free precession sequence with deep learning reconstruction
Source: J Cardiovasc Magn Reson. 2024 Jul 28;26(2):101069. doi: 10.1016/j.jocmr.2024.101069 (PMC11367510; doi:10.1016/j.jocmr.2024.101069)
Supplement: Supplementary file 1 — Supplementary material. [file mmc1.docx]

Supplementary

Supplementary Table 1. List of clinical protocols used per clinical indication. SAX short-axis; LAX long-axis; LVOT left ventricular outflow tract; LGE late gadolinium enhancement; IR inversion recovery; MRA magnetic resonance angiography; TI inversion time; STIR short tau inversion time inversion recovery; fat sat fat saturation; CAD coronary artery disease; C+ with contrast injection; NICMP non-ischemic cardiomyopathy

| **Clinical indication** | **Protocol** |
| --- | --- |
| **Hypertrophic cardiomyopathy** | - 3 plane localizers - Axial dark blood - Localizers for cine (3 slices in SAX and 2Ch, 4Ch views) - Cine (SAX stack, LAX: 2Ch, 3Ch, 4Ch views - T1/T2 mapping   ----- Contrast injection -----   - LVOT planimetry cine - Flow: LVOT below valve and perpendicular to jet - LGE scout: cine IR - LGE: SAX stack, LAX: 2Ch, 3Ch, 4Ch views |
| **Atrial fibrillation** | - 2 plane localizers - Axial dark blood - Localizers for cine (3 slices in SAX and 2Ch, 4Ch views) - Cine (SAX stack, LAX: 2Ch [3 slices], 3Ch, 4Ch)   ----- Contrast injection -----   - MRA: pulmonary veins - LGE (850 TI with 6 mm slice thickness, 2 mm gap): Atrial stack - LGE scout: cine IR - LGE: 2Ch (3 slices) |
| **Inflammation / Myocarditis** | - 3 plane localizers - Axial dark blood - Localizers for cine (3 slices in SAX and 2Ch, 4Ch views) - Cine (2Ch and 4Ch) - T1/T2 mapping - T2-STIR   ----- Contrast injection -----   - Cine (SAX stack and 3Ch) - LGE scout: cine IR - LGE: SAX stack, LAX: 2Ch, 3Ch, 4Ch views - LGE (fat sat): SAX (3 slices), LAX (4Ch view) |
| **Viability (CAD)** | - 3 plane localizers - Axial dark blood - Localizers for cine (3 slices in SAX and 2Ch, 4Ch views) - Cine (2Ch and 4Ch) - T1/T2 mapping   ----- Contrast injection -----   - Cine (SAX stack and 3Ch) - LGE scout: cine IR - LGE: SAX stack, LAX: 2Ch, 3Ch, 4Ch views - LGE scout: cine IR (null dark blood) - LGE (dark blood): SAX stack, LAX (4Ch view) |
| **Standard C+ exam (other NICMP)** | - 3 plane localizers - Axial dark blood - Localizers for cine (3 slices in SAX and 2Ch, 4Ch views) - Cine (2Ch and 4Ch) - T1/T2 mapping   ----- Contrast injection -----   - Cine (SAX stack and 3Ch) - LGE scout: cine IR - LGE: SAX stack, LAX: 2Ch, 3Ch, 4Ch views |
| **Healthy volunteer** | - 3 plane localizers - Axial dark blood - Localizers for cine (4 slices in SAX and 2Ch, 4Ch views) - T1/T2 mapping - Cine (SAX stack, LAX: 2Ch, 3Ch, 4Ch views) |

Supplementary Table 2. Comparison of measured functional parameters between ASSET bSSFP cine and Sonic DL bSSFP cine using SAX stack images. All volumes were indexed to height. Statistical significance was considered when p < 0.05. Values are presented as means ± standard deviation. ASSET array coil spatial sensitivity encoding; bSSFP balanced steady-state free precession; DL deep learning; LVEDV left ventricular end-diastolic volume; LVESV left ventricular end-systolic volume; LVEF left ventricular ejection fraction; LVM left ventricular mass; RVEDV right ventricular end-diastolic volume; RVESV right ventricular end-systolic volume; RVEF right ventricular ejection fraction; r correlation coefficient.

| **Parameters** | **LVEDV (mL/cm)** | **LVESV (mL/cm)** | **LVM (g/cm)** | **RVEDV (mL/cm)** | **RVESV (mL/cm)** |
| --- | --- | --- | --- | --- | --- |
| ASSET bSSFP | 0.83 ± 0.215 | 0.348 ± 0.189 | 0.715 ± 0.222 | 0.719 ± 0.223 | 0.273 ± 0.125 |
| Sonic DL bSSFP | 0.814 ± 0.207 | 0.349 ± 0.2 | 0.756 ± 0.233 | 0.719 ± 0.203 | 0.272 ± 0.123 |
| ASSET – Sonic DL | 0.016 ± 0.059 | -0.001 ± 0.048 | -0.039 ± 0.072 | 0.00002 ± 0.128 | -0.0002 ± 0.042 |
| p-value | 0.0426 | 0.8153 | 8.93⋅10^-5^ | 0.999 | 0.974 |
| r | 0.961 | 0.971 | 0.952 | 0.824 | 0.942 |

Supplementary Table 3. Comparison of measured functional parameters between ASSET bSSFP cine and Sonic DL bSSFP cine stratified by disease type. The sample size for each patient group is: atrial fibrillation (n = 38), suspected myocarditis (n = 23), suspected or known coronary artery disease (CAD) (n =13), hypertrophic cardiomyopathy (HCM) (n = 9), other non-ischemic cardiomyopathy (NICMP) (n = 10), and healthy volunteer (n = 15). Statistical significance was considered when p < 0.05. Values are presented as means ± standard deviation. ASSET array coil spatial sensitivity encoding; bSSFP balanced steady-state free precession; DL deep learning; LVEDV left ventricular end-diastolic volume; LVESV left ventricular end-systolic volume; LVEF left ventricular ejection fraction; r correlation coefficient.

|  |  | ASSET | Sonic DL | ASSET – Sonic DL | p-value | r |
| --- | --- | --- | --- | --- | --- | --- |
| Atrial Fibrillation | LVEDV (mL) | 144.7 ± 42.6 | 141.5 ± 40.7 | 3.17 ± 16.5 | 0.27 | 0.92 |
|  | LVESV (mL) | 51.4 ± 22.1 | 54.3 ± 22.8 | -2.9 ± 9.98 | 0.10 | 0.90 |
|  | LVEF (%) | 64.7 ± 12.8 | 61.6 ± 13.3 | 3.11 ± 5.56 | 0002 | 0.91 |
| Suspected Myocarditis | LVEDV (mL) | 158.2 ± 39.1 | 150.7 ± 36.3 | 7.54 ± 15.37 | 0.04 | 0.92 |
|  | LVESV (mL) | 64.1 ± 37.6 | 66.4 ± 41.1 | -2.27 ± 8.93 | 0.27 | 0.98 |
|  | LVEF (%) | 61.2 ± 15.3 | 57.7 ± 20.1 | 3.55 ± 7.54 | 0.049 | 0.94 |
| Suspected CAD | LVEDV (mL) | 180.0 ± 41.1 | 178.4 ± 40.7 | 1.57 ± 11.65 | 0.68 | 0.96 |
|  | LVESV (mL) | 103.5 ± 42.6 | 107.1 ± 47.7 | -3.55 ± 13.35 | 0.42 | 0.96 |
|  | LVEF (%) | 42.6 ± 19.0 | 41.3 ± 18.6 | 1.36 ± 9.37 | 0.66 | 0.88 |
| HCM | LVEDV (mL) | 111.2 ± 23.2 | 117.5 ± 29.1 | -6.22 ± 14.97 | 0.28 | 0.86 |
|  | LVESV (mL) | 30.7 ± 13.2 | 29.1 ± 10.4 | 1.61 ± 8.18 | 0.59 | 0.78 |
|  | LVEF (%) | 72.3 ± 10.6 | 75.0 ± 7.5 | -2.71 ± 6.94 | 0.31 | 0.76 |
| NICMP | LVEDV (mL) | 160.3 ± 58.6 | 157.9 ± 62.0 | 2.45 ± 11.78 | 0.53 | 0.98 |
|  | LVESV (mL) | 60.2 ± 47.2 | 65.7 ± 45.7 | -5.57 ± 9.97 | 0.11 | 0.98 |
|  | LVEF (%) | 65.9 ± 15.7 | 61.5 ± 11.9 | 4.47 ± 5.56 | 0.03 | 0.95 |
| Healthy volunteer | LVEDV (mL) | 143.7 ± 37.1 | 139.8 ± 34.8 | 3.84 ± 8.76 | 0.11 | 0.97 |
|  | LVESV (mL) | 41.1 ± 17.9 | 42.9 ± 19.5 | -1.77 ± 6.55 | 0.31 | 0.94 |
|  | LVEF (%) | 72.1 ± 6.6 | 70.1 ± 7.5 | 1.93 ± 3.67 | 0.06 | 0.87 |


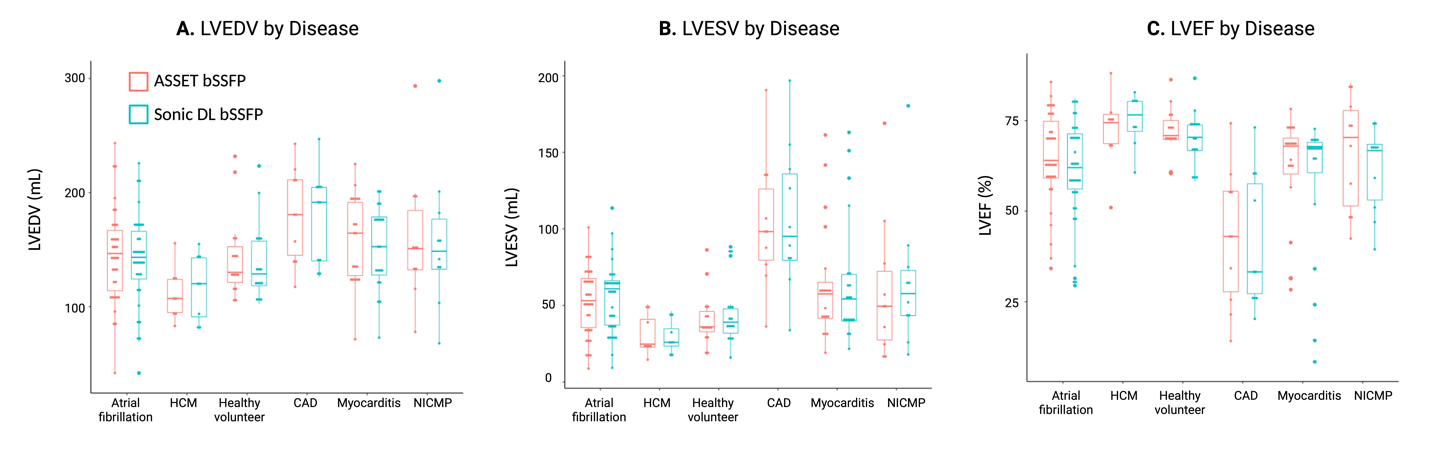


Supplementary Figure 1. Box-plots comparing volumetry and function as assessed by ASSET and Sonic DL stratified by disease type. The sample size for each patient group is: atrial fibrillation (n = 38), suspected myocarditis (n = 23), suspected or known coronary artery disease (CAD) (n =13), hypertrophic cardiomyopathy (HCM) (n = 9), other non-ischemic cardiomyopathy (NICMP) (n = 10), and healthy volunteer (n = 15). (A) LVEDV. (B) LVESV. (C) LVEF. ASSET array coil spatial sensitivity encoding; bSSFP balanced steady-state free precession; DL deep learning; LVEDV left ventricular end-diastolic volume; LVESV left ventricular end-systolic volume; LVEF left ventricular ejection fraction.
